# Hernia and Cancer: The Points Where the Roads Intersect

**DOI:** 10.3389/fsurg.2019.00019

**Published:** 2019-04-05

**Authors:** Hakan Kulacoglu, Ferdinand Köckerling

**Affiliations:** ^1^Ankara Hernia Center, Ankara, Turkey; ^2^Department of Surgery, Centre for Minimally Invasive Surgery, Vivantes Klinikum, Berlin, Germany

**Keywords:** hernia, hernia repair, mesh, cancer, chemotherapy, radiotherapy, surgical site infection

## Abstract

**Introduction:** This review aimed to present common points, intersections, and potential interactions or mutual effects for hernia and cancer. Besides direct relationships, indirect connections, and possible involvements were searched.

**Materials and Methods:** A literature search of PubMed database was performed in July 2018 as well as a search of relevant journals and reference lists. The total number of screened articles was 1,422. Some articles were found in multiple different searches. A last PubMed search was performed during manuscript writing in December 2018 to update the knowledge. Eventually 427 articles with full text were evaluated, and 264 included, in this review.

**Results:** There is no real evidence for a possible common etiology for abdominal wall hernias and any cancer type. The two different diseases had been found to have some common points in the studies on genes, integrins, and biomarkers, however, to date no meaningful relationship has been identified between these points. There is also some, albeit rather conflicting, evidence for inguinal hernia being a possible risk factor for testicular cancer. Neoadjuvant or adjuvant therapeutic modalities like chemotherapy and radiotherapy may cause postoperative herniation with their adverse effects on tissue repair. Certain specific substances like bevacizumab may cause more serious complications and interfere with hernia repair. There are only two articles in PubMed directly related to the topic of “hernia and cancer.” In one of these the authors claimed that there was no association between cancer development and hernia repair with mesh. The other article reported two cases of squamous-cell carcinoma developed secondary to longstanding mesh infections.

**Conclusion:** As expected, the relationship between abdominal wall hernias and cancer is weak. Hernia repair with mesh does not cause cancer, there is only one case report on cancer development following a longstanding prosthetic material infections. However, there are some intersection points between these two disease groups which are worthy of research in the future.

## Introduction

Cancer is one of the leading causes of major problems in human health worldwide (1). The estimated global numbers of new cancer cases and cancer deaths in 2018 are 17 million and 9.5 million, respectively (2). It is claimed “if a person lives long enough, he or she will suffer cancer eventually” (3). The debate continues about cancer development, whereby intrinsic and extrinsic risk factors like genetic, lifestyle, and environmental factors are implicated (4).

Abdominal wall hernias are very frequent surgical diseases. Groin hernia repair constitutes the largest group, accounting for ~70% of all hernia repairs (5, 6). Globally, more than 20 million patients undergo groin hernia repair every year (7). By a simple calculation, the estimated annual total number for all types of hernia repairs worldwide may reach 30 million annually. In terms of etiology, hernias are classified as either primary or secondary hernias. Primary hernias may be congenital or acquired (8–11). Unlike cancers, hernias are benign conditions and cure without recurrence is possible in almost 95% of cases, especially for groin hernias (12, 13), although the recurrence rates are higher for ventral hernias (14–16). Morbidity is also very low and the mortality rate is almost zero after hernia repairs except for emergency repairs, especially in the elderly (17–20).

Two different medical entities may be encountered with regard to certain points. These potential intersection points may share a common etiology, causing, initiating, promoting, or aggravating each other as well as the responsiveness to the same or similar treatment methods. Moreover, it should be established whether a treatment method used for one of these two conditions could have detrimental effects on the other one. In this review we aimed to search common points, intersections and potential interactions or mutual effects for hernia and cancer. Besides direct relationships, indirect connections and possible involvements were searched.

## Materials and Methods

**Figure 1:**
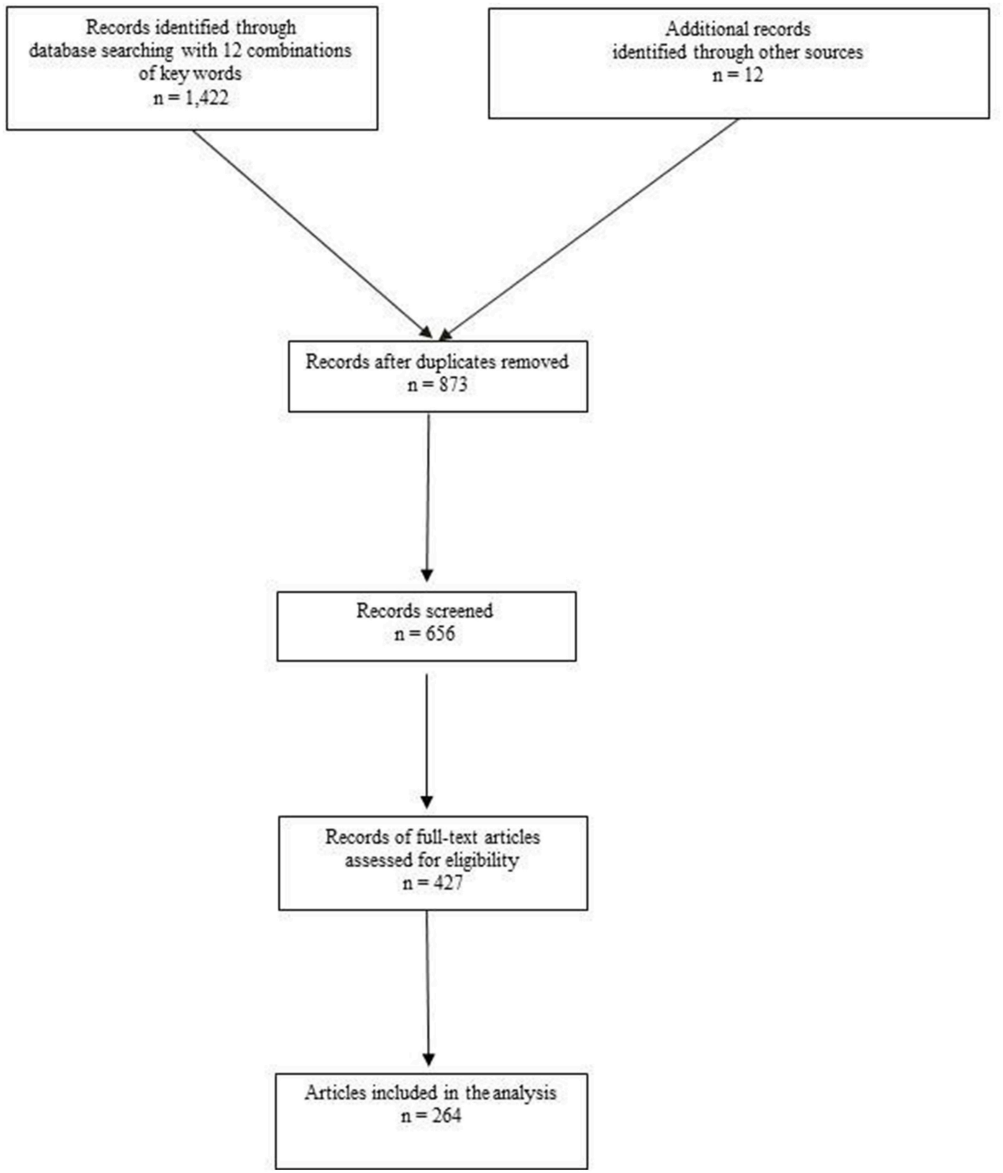
Flowchart of information through the different phases of analysis.

A literature search of PubMed database was performed in July 2018 as well as a search of relevant journals and reference lists. The following search terms were used to reach the potential direct and indirect relationships between hernia and cancer: “inguinal hernia and cancer,” “incisional hernia and cancer,” “umbilical hernia and cancer,” “paraumbilical hernia and cancer,” “femoral hernia and cancer,” “obturator hernia and cancer,” “lumbar hernia and cancer,” “Spigelian hernia and cancer,” “trocar site hernia and cancer,” “hernia mesh cancer,” “prosthetic mesh cancer,” “chemotherapy and hernia,” “chemotherapy and incisional hernia,” “radiotherapy and hernia,” “radiotherapy and incisional hernia.” The total number of screened articles was 1,422. Some articles were found in multiple different searches. A last PubMed search was performed during manuscript writing in December 2018 to update the knowledge. Eventually 427 articles with full text were evaluated, and 264 included, in this review (Figure 1).

## Results

In the literature there is no evidence for a possible common etiology for abdominal wall hernias and any cancer type. The two different diseases had been found to have some common points in the studies on genes, integrins, and biomarkers, however, to date no meaningful relationship has been identified between these points. There is also some, albeit rather conflicting, evidence for inguinal hernia being a possible risk factor for testicular cancer.

As a secondary type, incisional hernias were quite frequent following abdominal surgery for cancer, not only after laparotomy but also after a laparoscopic approach. Incisional hernias after cancer surgery do not cause an increase in mortality, but incarceration is associated with a worse outcome.

Neoadjuvant or adjuvant therapeutic modalities like chemotherapy and radiotherapy may cause postoperative herniation with their adverse effects on tissue repair. Certain specific substances like bevacizumab may cause more serious complications and interfere with hernia repair.

There are only two articles in PubMed directly related to the topic of “hernia and cancer.” One of these is a retrospective series of 1,894 patients undergoing hernia repair with mesh (21). These patients were compared to a cohort control group undergoing cholecystectomy. The authors claimed that there was no association between cancer development and hernia repair with mesh. The other article reported two cases of squamous-cell carcinoma developed secondary to longstanding mesh infections (22). Ten articles on the relationship between prosthetic materials and cancer development were also evaluated.

All the objective findings and the clues for potential relationships were given in Table 1.

**Table 1:** Objective findings and the potential relationships for hernia and cancer.

Common etiologic factor	No data on a common etiology for abdominal wall hernias and any types of cancer.
	Certain factors (e.g., obesity, smoking, estrogens) may play role in both hernia and cancer development in separate ways.
Role of genetics: genes, biomarkers, etc.	Genetic factors may have a role in the etiology of both cancer and abdominal wall hernias.
	Positive family history may of importance in both.
	Studies on some biomarkers may provide some clues in the future.
Blockage of integrins	Blockage with recombinant RGD disintegrin may have protective effect on both tumor progression and incisional hernia development.
Inguinal hernia as a possible risk factor for testicular cancer	Data exist for a possible correlation between childhood inguinal hernia and testicular cancer.
Do hernial diseases protect against cancer	No objective findings.
Prognosis of patients with incisional hernia	No effects unless incarceration develops, however health-related quality of life is worse.
Adverse effects of chemotherapy and/or radiation therapy	They may cause postoperative herniation by adverse effects on tissue repair. Certain specific substances like bevacizumab may complicate hernia repairs.
Can mesh cause cancer	No direct causation relationship.
	Case report for cancerogenesis on a longstanding surgical site infection after mesh repair.
	Data exist for gynecologic sling procedures possibly due to chronic irritation or infection.

## Discussion

The findings obtained in the present search are now discussed below together with some other relevant publications under several separate subheadings.

### Any Common Etiologic Factor?

To date, there are no data on a common etiology for abdominal wall hernias and any types of cancer. Maybe we need to mention obesity as a common risk factor for development of cancer and hernias. Metabolic changes in obesity may contribute directly or indirectly to cancer (23). There is evidence from epidemiologic and preclinical studies for a relationship between obesity and breast, prostate, esophageal, colorectal, pancreatic, liver, and endometrial cancer as well as renal cell carcinoma (24, 25). Some studies stressed the role of visceral obesity defined by waist circumference instead of high body mass index (BMI) (26–30).

Obesity has also been implicated as a predisposing factor for abdominal wall hernias. Although high BMI has been found to be associated with a low incidence of inguinal hernias in both men and women (31–33), obesity is thought to increase the incidence of acquired ventral hernias (34). Obesity may also give rise to trocar site hernia development (35). Moreover, higher recurrence rates of different types of hernias after repair have been reported in obese patients (36–38). It has also been reported that, unlike elevated BMI (39), visceral obesity measured by visceral fat volume, subcutaneous fat volume, total fat volume, and waist circumference is strongly associated with incisional hernia after colorectal surgery (40). Similarly, higher subcutaneous fat deposition at the level of the umbilicus has been reported as a risk factor for incisional hernia development following surgery for colorectal cancer (41). Waist circumference is also an independent risk factor for parastomal hernia development (42). In addition, obesity has been implicated as a risk factor for recurrence after incisional hernia repair (43) and umbilical repair (37).

In the light of existing publications, although obesity does not seem to be a common etiologic factor for cancer and hernias, it constitutes an intersection point for the two diseases.

Smoking has been reported to be associated with not only lung cancer but also with some other cancers (44–48). It has also negative effects on survival, and smoking-cessation programs could assure a longer life span (49–52). The effect of smoking on hernia development is controversial. On the one hand, it has been reported that smokers may develop more groin hernias than non-smokers (53). Read showed that smokers have a higher circulating serum elastolytic activity in comparison with non-smokers (54). Serum elastolytic and protease substances were higher in the blood of patients with inguinal hernia than in controls (55). Jansen et al. identified smoking as a risk factor for early onset of hernia disease (56). Nevertheless, a community-based study from Sweden demonstrated that smokers had a 26% lower risk of groin hernia (33). Another observational study from Sweden again revealed that smoking was not a risk factor for an increase in groin hernia repair (57). However, smoking is a risk factor for incisional hernia, and it has been shown that smokers have a 4-fold higher risk of incisional hernia development (58). It has also been reported that smoking is a risk factor for recurrence after groin hernia repairs, possibly due to abnormal connective tissue metabolism in smokers (59). The mechanism for recurrence in smokers may be a systemic protease/antiprotease imbalance that might cause fascial degeneration, and adversely affect wound healing (60). On the other hand, smoking has not been found to be a risk factor for recurrence of umbilical (36) or inguinal hernias (56).

Like obesity, smoking does not seem to be a common etiologic factor for cancer and hernias; moreover, the intersection points are more obscure.

Estrogen is a well-established risk factor for many types of cancer, particularly breast and endometrial cancer (61). The primary mediators of estrogen action are estrogen receptor ERα and ERβ. The oncogenic effects of estrogen are mainly via ERα-mediated transcriptional activation of genes that play key roles in cell proliferation or reduce apoptosis (61, 62). Today, the effect of estrogen on tendons and ligaments is not well-understood (63). Estrogen may inhibit the local inflammatory response and impair wound healing (64). In a clinical study on fibroblasts retrieved from recurrent hernia patients, it has been shown that estradiol has no influence on the impaired type I to type III collagen ratio (65). As early as 1960, Hazary and Gardner reported that male mice given estrogen for prolonged periods developed inguino-scrotal hernias (66). A very recent study by Zhao et al. has also revealed that the shift from androgen to estrogen causes inguinal hernia in male mice, and this effect could be entirely prevented by an aromatase inhibitor (67). Tamoxifen is a commonly used selective estrogen receptor modulator that has been used for hormonal treatment of breast cancer for a long time now (68, 69). Its side effects and potential risks have been well-studied in women (70). The potential side effects in men are infertility and gynecomastia (71). Ma et al. reported that tamoxifen might cause hernia development in mice by activating matrix metalloproteinase expression (72). These interesting findings create an intersection for hernia and cancer and future studies could provide more surprising results on this topic.

Regarding mutual causation, there is no evidence and even no argument about cancer development secondary to any hernia. However, as discussed below, a low-grade contention has been upheld suggesting that the prosthetic material used for hernia repair induced carcinogenesis. On the contrary, abdominal wall hernias may develop not because of the cancer itself but due to its consequences, for example increased intra-abdominal pressure secondary to an obstructive colon cancer or a large pelvic tumor may cause herniation. The necessity for colorectal cancer screening in patients with inguinal hernia will be discussed in an upcoming part of this review.

### Role of Genetics… Genes, Biomarkers and More

Genetic factors may have a role in the etiology of both cancer and abdominal wall hernias. The first studies on a genetic factor for hernias were published in the 1970s (73, 74). In 1994, Gong et al. suggested that most affected autosomal dominant inheritance with incomplete penetrance (75). They found that persons with indirect inguinal hernias might have inherited a gene, more frequently from their father. In the new millennium, genetic studies on inguinal hernias have gone much further. A Swedish study revealed that a positive family history of hernia surgery was a risk factor for abdominal wall hernias (76). Genetic studies from China revealed that DNA sequence variants (DSVs) might contribute to inguinal hernia development by changing the transcriptional activities of TBX1, TBX2, TBX3, Sirtuin 1, and GATA transcription factor 6 (GATA6) gene promoters (77–81). Some of these genetic alterations may also play a role as etiologic or prognostic factors in human cancers (82).

Jorgenson et al.'s genome-wide association study is worth mentioning here (83). They found four novel loci for the etiology of hernia development. These loci for inguinal hernia susceptibility are EFEMP1, WT1, EBF2, and ADAMTS6. Wang et al. demonstrated that EFEMP1 (epidermal growth factor-containing fibulin-like extracellular matrix protein 1) might enhance the expression of matrix metalloproteinase-2 (MMP-2) and promote the migration and invasion of osteosarcoma (84). The same group also reported that EFEMP1 promotes ovarian cancer cell growth and metastasis (85), and is a potential diagnostic biomarker for prostate cancer (86). WT1 gene, although it is a suppressor gene in kidney tumors, acts as an oncogene in leukemia, lung cancer, breast cancer, and glioblastoma (87).

Although we can see some intersections for hernia and cancer above, none of these findings means very much today. Therefore, it is not possible to make any statement about a possible etiologic factor. However, new clues could arise from newer genetic studies on abdominal wall hernias.

This has been an interesting field where researchers aimed to identify a particular biomarker for the prediction of abdominal wall hernia development. A recent Dutch study found that advanced glycation end products (AGEs) might be used as a biomarker for incisional hernia. Measurements based on the use of a Skin Auto Fluorescence (SAF) reader revealed that AGEs were significantly higher in patients with incisional hernia than in the control group (88). AGEs are harmful compounds generated by non-specific glycation of proteins and lipids which are related to cardiovascular diseases, arteriosclerosis, and neurodegeneration (89). Likewise, they may have a possible role in cancer initiation and progression (90). They have been thought to promote a breast cancer cell line by enhancing proliferation, invasion, and migration (91). On the other hand, it has been shown that AGE treatment of ER+ breast cancer cells altered ERα phosphorylation and promoted resistance to tamoxifen therapy (92). A recent experimental study with a mouse model revealed that advanced glycation end product N^ϵ^-carboxymethyllysine could promote progression of pancreatic cancer (93). Another animal study demonstrated that there might be a link between AGEs and lung cancer (89).

Decreased collagen I/III ratios are now a well-known state in incisional hernia patients. Rosch et al. by confirming this, demonstrated that c-myc levels were significantly elevated in patients with incisional hernia (94). c-MYC is among the most frequently affected genes in human cancers, and has a pivotal function in growth control, differentiation and apoptosis and its abnormal expression is associated with many tumors (95). Wang et al. reported that positive expression of C-myc resulted in shorter disease-free survival and increasing recurrence rates in triple negative breast cancer (96).

Calaluce et al. using microarray analysis, identified a distinct gene expression profile in patients with recurrent incisional hernia (97). They revealed an association between GREM1, a bone morphogenetic protein antagonist 1, and incisional hernia formation. GREM1 was underexpressed in skin and fascia of patients with recurrent hernia compared with control subjects.

Although GREM1 might promote carcinogenesis (98), its expression has also been shown to be associated with a good prognosis in gastric and colorectal cancers (99–101).

As presented above, while at least some direct and indirect evidence has been obtained for certain biomarkers in hernia formation and for the relationship between these markers and cancer, it is not possible to demonstrate a link between hernia formation and carcinogenesis. Further studies may or may not change the picture in the future.

An international, multicenter study group named ColoCare has been recruiting colorectal cancer patients to identify biologic markers to predict clinical outcomes (102). In one of their studies they researched predictive biomarkers for incisional hernia development after cancer surgery (103). Some proteins including RNA polymerase III transcription factor, calreticulin, estrogen receptor 1, and Harvey rat sarcoma viral oncogene homolog were found to be higher in cases of postsurgical incisional hernia. Furthermore, in gene set analysis, melanoma, bladder cancer, focal adhesion, pathways in cancer, etc. had a *p* < 0.05. Although there is a certain relationship between these proteins and cancerogenesis (104–106), this does not mean shared or similar etiological mechanisms for hernia development and cancer. In fact, many proteins and genes play complex roles in cancer biology; the same substance may have both promoter and inhibiter effects on different levels of cancerogenesis and metastatic disease.

### What About Blockage of Integrins?

Integrins are the main receptor proteins that cells use to bind to the extracellular matrix (107, 108). They consist of an α and αβ subunits. The α2β1 mediates keratinocyte adhesion to collagen I for epidermal wound healing (109). The αvβ3 integrin is overexpressed by platelets, endothelial cells, macrophages, and fibroblasts during wound healing. Inhibition of αvβ3 with antibody results in a decrease in the migration of these cells and angiogenesis (110, 111). Integrins are involved in many pathological conditions such as inflammation and tumor progression (112). Several studies revealed that the angiogenic and tumorogenic effects of αvβ3 could be prevented by a novel recombinant RGD disintegrin (DisBa-01) produced from a Brazilian snake (113, 114). This also works against metastasis (115, 116). Moreover, the blockade with DisBa-01 may be preventive against incisional hernia development. A recent animal study from Brazil demonstrated that DisBa-01 increased the number of macrophages and fibroblasts and no subject treated with this recombinant disintegrin developed incisional hernia (117). The authors concluded that this might be a promising therapeutic tool in wound healing and incisional hernia prevention. In fact, these findings may not only present a hope for lower incisional hernia rates in the future but may also reflect a common etiological factor for cancer and a type of abdominal hernias.

### Inguinal Hernia as a Possible Risk Factor for Testicular Cancer

Till today, no series has been published revealing a high incidence of any cancer type in patients with abdominal wall hernias, or vice versa. However, there are reports of a correlation between inguinal hernia and testicular cancer. Earlier reports on this topic were very close to implicating childhood inguinal hernia in the development of testicular tumors. In 1976, Morrison reported that the testicular cancer risk was almost three times higher in men who had had an inguinal hernia than in those who had not, with no association seen between the side of hernia and side of tumor (118). Coldman et al. confirmed this relationship in a series of 128 patients with testicular seminoma (119). They found that the probability of testicular tumor was higher in patients with inguinal hernia. That effect was even greater in the presence of concomitant cryptorchidism. In fact, congenital inguinal hernia is secondary to incomplete obliteration of the processus vaginalis, whereas an undescended testis is another aspect of the same process. This might indicate a common etiology. A UK study also reported that inguinal hernia was a risk factor for testicular cancer; hernia repair before the age of 15 years was a risk factor for seminoma (120). Likewise, a USA study reported that a hernia operation after age 7 years presented a significantly elevated risk of testicular cancer (121). In 1994, the United Kingdom Testicular Cancer Study Group reconfirmed that testicular cancer may be associated with inguinal hernia (122). This risk is higher when the age of diagnosis is <15 years. One year later, Gallagher et al. reported that inguinal hernia requiring surgery was a risk factor for testicular cancer (123). Nevertheless, just after, two case-control studies from Denmark stated that inguinal hernia was not a risk factor for testicular cancer in the absence of cryptorchidism or testicular atrophy (124).

When we evaluated more recent data, a study from the Slovak Republic revealed that inguinal hernia was one of the most prominent risk factors for testicular cancer (125). More than 11% of the testicular cancer patients also had a history of inguinal hernia in the report of the International Testicular Cancer Linkage Consortium (126). A recent meta-analysis showed that previous inguinal hernia is a consistent factor with an increased risk of testicular cancer (1·63, 1·37–1·94) (127). The report of the ESMO Consensus Conference on testicular germ cell cancer stressed this relationship (128). As a bottom line, we can say that the relationship between the testicular tumors and inguinal hernia is definitely a point where the roads of cancer and hernia intersect.

### Read's Theory: Do Hernial Diseases Protect Against Cancer?

Raymond C. Read, the father of the term “herniologist,” hypothesized that the circumstances that cause abdominal wall hernias could have a protective effect against cancer development (129). Starting from the changes in collagen metabolism in patients with hernias, he stressed the biochemical evidence available for the extracellular matrix (ECM) in patients with colonic diverticulosis. Both hernia patients and diverticulosis patients have a lower collagen type I/type III ratio. He cited Schumpelick et al. studies that revealed a significantly reduced incidence of diverticula in patients with colon cancer (130–132). Indeed, an Aachen group scrutinized this subject and published several related papers, however, they never mentioned any pathways involving hernia disease. Later, Filik and Biyikoglu wrote a letter to Read's editor and agreed with him on a possible preventive effect of diverticulosis against colonic cancer (133). Read, in his response to this letter, stressed the importance of the ECM again by citing the work of Ghajar and Bissell's study on mammary gland tumorigenesis in which the authors described the ECM as “more than just a scaffold.” This study from the Life Sciences Division of Lawrence Berkeley National Laboratory Interaction stated that the changes in ECM composition and structure disrupt tissue organization, and interaction with the ECM influences malignant progression (134). Nevertheless, no objective findings have been presented to date about the protective effects of hernia disease against cancer.

Another topic related to the above one is the difference in incisional hernia rates after surgery for diverticulitis and cancer. Pogacnik et al. reported that the incidence of incisional hernia following sigmoidectomy for diverticulitis was significantly higher in comparison with cancer surgery (135). The authors speculated that several factors including collagen tissue disorder might play a role for this finding. Prasad et al. criticized this paper in a letter by pointing to the fact that diverticulosis is already present in almost half of patients with colon cancer and this may create a bias when interpreting the result of the study by Prasad et al. (136). Very recently, Tang et al. reported that the incisional hernia rate was higher in patients undergoing surgery for diverticular disease than in patients with colorectal cancer (137). They did not give any reason for this difference. This topic seems to need more work to permit a solid conclusion.

### Abdominal Surgery for Cancer as an Etiologic Factor for Hernia Development

A certain number of laparotomies results in incisional hernias. Therefore, abdominal surgery for cancer is the main cause of herniation through laparotomy incision. Herniation can be seen at the port sites used for laparoscopic surgery for cancers (138). For example, 5–50% of the patients who undergo open surgery for colorectal cancer develop an incisional hernia (139, 140). Stoma creation is an important part of colorectal cancer surgery. Herniation through the stoma site, the entity named parastomal hernia, is not rare (141, 142). Its treatment is also challenging (143). These issues will be addressed as specific topics in separate parts of this review. Surgical treatment of prostate cancer may not only result in incisional hernia but also in inguinal hernia (144, 145). This will also be discussed as a separate topic later on.

### Does Incisional Hernia Development Affect Cancer Prognosis?

This is an interesting question. There is only one study that evaluated this subject. Jensen et al. studied the impact of incisional hernia on mortality in patients who had undergone colorectal cancer surgery (146). Seven per cent of 9,214 patients were diagnosed with a herniation after a median 6.4 years of follow-up. This diagnosis was not linked to an increase in mortality but incarceration was associated with a worse outcome. The authors reported a 7.7% 30-day mortality rate after incarcerated incisional hernia in comparison with 0.85 for uncomplicated ones.

A very recent national prospective cohort study from Denmark focused on the impact of incisional hernia on health-related quality of life after cytoreductive surgery and hyperthermic intraperitoneal chemotherapy (147). CRS + HIPEC do not increase the risk of incisional hernia as measured within 12 months postoperatively, but when developed, incisional hernia adversely affected health-related quality of life. Patients with incisional hernia suffered a reduction in work and daily activities because of the effects on their physical and psychological health. This study did not provide any information on differences in prognosis or mortality rates.

### Adverse Effects of Treatment Methods

When it comes to treatment there are no therapeutic methods to which both hernia and cancer respond well. Surgical intervention is the treatment of choice for both entities but there is no other common point. However, there are always possibilities that the treatment method used for one disease could have adverse effects on the other.

Tamoxifen is a tool for hormonal therapy for breast cancer. Hernia development due to tamoxifen use in mice, by activating matrix metalloproteinase expression, is discussed above.

Chemotherapy and radiotherapy for cancers have well-known adverse effects on wound healing (148). However, these effects have been mostly evaluated in animal studies (149–152). Human and animal studies revealing conflicting results were obtained regarding the collagen content of the irradiated tissues and the ratios for different collagen types (153–161). It has also been demonstrated that radiation might cause terminal differentiation of human fibroblasts (162). Interestingly, any given specific chemotherapeutic agent may impair wound healing, but a combination of some others can reverse this effect (152).

The evidence available for human beings is rather sparse. The studies are retrospective because of the nature of the subject. There is a paucity of data, especially about the effects of radiotherapy on abdominal wall hernia surgery. A relatively large study from MD Anderson Cancer Center revealed that previous radiotherapy did not affect the outcomes after midline abdominal wall reconstruction with preperitoneal, intraperitoneal, or retromuscular mesh placement (163). Hernia recurrence rates and time to recurrence were similar for patients who received or did not receive radiotherapy. Likewise, mesh infection, mesh removal, and reoperation rates did not differ.

Neoadjuvant chemotherapy has been shown to be related to delayed healing and wound complications after abdominoperineal resection (164). Early postoperative intraperitoneal chemotherapy reduces collagen accumulation in human subjects (165). Adjuvant chemotherapy is also thought to impair postoperative wound healing, hence its administration is generally delayed until complete recovery from surgery. However, there is some conflicting evidence that chemotherapeutic agents might not adversely affect wound healing in the early period (166).

The effects of chemotherapy and/or radiotherapy on incisional hernia development have not been studied widely or specifically. Rettenmaier et al. found that radiotherapy and treatment with bevacimuzab and liposomal doxorubicin were significant factors for accelerated incisional hernia development in gynecologic cancer patients (167). Recently, Itatsu et al. reported that preoperative chemotherapy was one of the independent factors for incisional hernia development (168). Conversely, Fazekas et al. stated that neither preoperative/adjuvant chemotherapy nor preoperative radiotherapy was a risk factor for incisional hernia following ileostomy reversal in colorectal cancer patients (169). Similarly, Oliphant et al. found that adjuvant chemotherapy might cause more stoma complications after surgery for colorectal cancer, but there was no rise in the frequency of paratomal hernia (170). Likewise, Struller et al. claimed that hyperthermic intraperitoneal chemotherapy along with cytoreductive surgery was not associated with an increased risk of incisional hernia development (171).

Bevacizumab is an antiangiogenic agent and is used in combined chemotherapy as a monoclonal antibody. It provides promising results even in resistant malignancies and metastatic diseases (172, 173). Its efficacy generally comes with good safety (174) but it may increase the incidence of wound-healing complications (175), and even serious intra-abdominal complications like intestinal fistula have also been reported (176–178). Eriksen and Bulut reported a case of enterocutaneous fistula after perineal hernia repair with biological mesh (177). The patients had undergone abdominoperineal resection and been put on a chemotherapy protocol including bevacizumab 6 weeks earlier. It was reported that pelvic floor reconstruction with biological mesh was superior to synthetic mesh use regarding the complications like erosion and infection (0 vs. 4%, and 0 vs. 13%) (179). Therefore, Eriksen and Bulut claimed that enterocutaneous fistula was an adverse effect of bevacizumab (177). Initial Safety Report of NSABP C-08 study revealed that wound complications including symptomatic incisional hernia were more common after 1 year in the patients given bevacizumab (180). Another safety study among patients receiving neoadjuvant chemotherapy for advanced stage peritoneal and ovarian cancer found that one out of 11 patients who received a protocol including bevacizumab developed incisional hernia following interval debulking surgery, whereas no herniation was recorded in 13 patients who did not receive this agent (181). Although the incisional hernia rates were 9 vs. 0%, the groups were very small and it is not possible to report a significant difference.

One interesting case related to bevacizumab use was reported from Greece (182). A patient receiving chemotherapy plus bevacizumab for colorectal liver metastasis had developed an incarcerated recurrent incisional hernia 2 weeks after the last administration of the protocol. Despite the risk of impaired wound healing and infection, an emergency hernia repair had been decided. The surgical team had used a biological mesh for repair. The patient did not develop recurrence after 1 year postoperatively.

Corticosteroids are known to have detrimental effects on wound healing and collagen metabolism (183–185). In a review article, Gopal and Warrier reported that long-term steroid therapy may be involved in recurrence after groin hernia repair (60).

They are sometimes involved in chemotherapy protocols for cancer patients. Therefore, combination chemotherapy protocols including corticosteroids may have some effects on incisional hernia development after abdominal cancer surgery or outcomes of mesh-based repair in cancer patients. However, to date there are no precise data in the literature on this particular subject. Recently, a prospective randomized study was registered to research the effects of preoperative administration of high-dose glucocorticoid to improve recovery and decrease the length of hospital stay in patients undergoing abdominal wall reconstruction. This interesting study is expected to document the impact of glucocorticoids on wound healing in hernia patients, as a secondary outcome measure (186).

In fact, the use of mesh for hernia repair in patients receiving chemotherapy is an important issue. However, to date the surgical site infection rates and recurrence risk have not been studied well. Immunosuppression due to chemotherapeutic agents or malignancy itself can not only increase the infection risk but may also result in higher recurrence rates because of impaired inflammatory response to tissue integration and scar formation. A retrospective series revealed that surgical site occurrence rates after incisional hernia repair with biological mesh did not differ between the groups receiving or not receiving chemotherapy (187). However, some problems might arise when synthetic meshes are used instead of expensive biological meshes. A recent controlled clinical study among patients undergoing chemotherapy for colon or ovarian cancer found that significantly more complications were associated with synthetic mesh use in comparison with biological meshes (188). A case of synergistic gangrene after incisional hernia repair with large porous polypropylene mesh in inlay position was also presented (189). The patient's body mass index was 50 and his primary disease was chronic lymphoblastic leukemia. Surprisingly, mesh salvage was achieved after observing healthy granulation tissue over the mesh following extensive surgical debridement.

Some groups have used biological meshes for abdominal wall closure after cytoreductive surgery and hyperthermic intraperitoneal chemotherapy. They reported that this technique might prevent subsequent incisional development, but at a cost of high infection rates and even enterocutaneous fistula formation (190, 191).

Another pharmacologic agent that deserves to be mentioned in this review is rapamycin. It has both immunosuppressant and anti-tumor properties (192). An experimental study from Germany revealed that rapamycin inhibits vascularization around synthetic mesh (193). This effect was found to be dose dependent. Animals receiving rapamycin showed markedly reduced amount of collagen fibers in the granulation tissue. The authors recommended against the use of rapamycin in patients undergoing incisional hernia repair.

### Hernia Meshes: Can They Cause Cancer?

Cancer is a group of diseases characterized by uncontrolled growth and spread of abnormal cells. It develops over time with many molecular changes that eventually produce malignancy (194). Both intrinsic and extrinsic factors may play a role (195–197). Certain chemicals have been implicated as carcinogens. Chemical carcinogens may cause DNA damage and contribute to cancer development (198, 199). It was also shown that solid objects/materials may initiate malignancies at the site of use under certain circumstances. The development of these tumors is thought to be dependent on the physical nature rather than the chemical nature of the material used. This was first reported by Turner in 1941 (200). Sarcoma developed around a plastic disk used as a subcutaneous implant in an animal study. In another experimental animal study, Zollinger reported that tumor induction could be produced by foreign bodies without the involvement of chemical carcinogens (201). He showed that tumors might arise as a result of the proliferative irritant effect of the plastic capsule on renal tissue. Nevertheless, when plastic fragments were implanted subcutaneously no tumor development was observed. This type of localized tumorigenic effect has become known as solid-state or foreign-body carcinogenesis. Many other materials like Dacron, nylon, polyethylene, polyvinyl chloride, silk, silastic, Teflon have been demonstrated to induce foreign-body carcinogenesis (202). There might be a latent period before the appearance of the tumors which could be correlated with the length of time necessary for polymer breakdown and the slow rate of release of the breakdown products, since carcinogenic activity would then be a cumulative function of this degradation (202). The development of implantation site tumors has also been correlated with the size of the implant: the greater the surface area of the implant, the higher the tumor incidence (203).

Patients for whom mesh repair of abdominal wall hernias is indicated sometimes ask the surgeons whether hernia meshes can cause cancer (204). The ideal mesh was described as being chemically inert and devoid of a carcinogenic effect (205). To date, there is no strong evidence about the role of prosthetic materials in cancer development as an extrinsic factor. In 2002, after reviewing the studies on the analyses of molecular markers of proliferation and the modulation of heat shock proteins, Ghadimi et al. stated that there was no evidence suggesting that human beings develop malignant tumors due to mesh (206). In 2014, Moalli et al. concluded that the potential carcinogenic risk presented by polypropylene meshes was extremely low (207). The only evidence of a relationship between cancer and polypropylene meshes has been obtained from animal studies and the risk of carcinogenesis in humans has not been confirmed.

Very recently, Chughtai et al. revealed that mesh-based hernia repair was not associated with an increased risk of subsequent development of cancer in men (21). They compared patients who underwent hernia repair with a group of control patients who underwent cholecystectomy or total knee replacement. Cancer development rates did not differ between the patient groups. To date, no study has addressed the same subject in the women, but polypropylene meshes used for gynecologic procedures have been implicated as a potential risk factor for cancer development in women. The suggested mechanism is based on an inflammatory reaction to the meshes and the subsequent oxidative process and free radical release, such as hydrogen peroxide (208, 209). Furthermore, some potentially toxic substances released from the degraded mesh can cause more complex inflammatory reactions and cascades (208). Bacterial contamination and proliferation may also contribute to further tissue reactions.

Birolini et al. reported that it was not the mesh itself but the mesh-related chronic infection and inflammation that can cause cancer (22). They recorded two cases of squamous-cell carcinoma in the abdominal wall secondary to long-term mesh infection. The mechanism is probably similar to the Marjolin's ulcers of burns. The authors resected the tumor and the affected bowel and, interestingly, reconstructed the abdominal wall using polypropylene mesh again. The prognosis was very poor. The authors concluded that infected meshes must be excised completely without delay. Apart from these two cases reported by Birolini et al. there is no single one case of malignancy related to mesh repairs for abdominal wall hernias in the literature.

Surgery involving the use of prosthetic mesh is not limited to the treatment of abdominal wall hernias. Similar materials are also used in urogenital surgical procedures, especially in sling techniques for stress urinary incontinence (210). Recently a case of clear cell carcinoma associated with delayed extrusion of mid-urethral polypropylene tape was reported 10 years after insertion (211). The authors' explanation for malignant degeneration was a possible mutation due to longstanding chronic tissue inflammation caused by mesh as a foreign body. Two interesting cases were reported by Ahuja et al. (212) of female patients who had undergone urogenital sling procedure with mersilene and polypropylene meshes. Cancer was diagnosed by endoscopic biopsy, and the patients underwent radical cancer surgery along with complete mesh excision over the sacrum. Both tumors were attached to the meshes, therefore the authors thought that chronic irritation due to mesh could be a contributory factor for cancerogenesis. King et al. studied this subject in a group of 2,361 patients who underwent polypropylene mid-urethral sling placement (213). They detected just one case of bladder cancer and one case of vaginal cancer, and no sarcomas after the sling procedure. The rate was practically zero per cent. A recent editorial by Goldman and Dwyer stated that surgeons should continue to reassure their patients of the low risk of cancer after the use of polypropylene grafts (214).

### What Is Later On

This review has been planned to comprise several parts. The present one is an overview of general issues about hernia and cancer. The forthcoming parts will be on specific types of cancers like urogenital cancers (oriented on prostate cancer), cancers of gastrointestinal tract (oriented on colorectal cancer) and oncologic surgery that compromise the integrity of the abdominal wall by direct or indirect ways (primary abdominal wall tumors and reconstructive surgery for breast cancer patients), primary or metastatic cancers that mimic hernias, and miscellaneous subjects about hernia and cancer intersections.

## Author Contributions

HK: study design, literature search, concept of manuscript, manuscript writing, critical overview. FK: concept of manuscript, critical overview, final submission.

### Conflict of Interest Statement

The authors declare that the research was conducted in the absence of any commercial or financial relationships that could be construed as a potential conflict of interest.
